# Comparison of Porcine Airway and Intestinal Epithelial Cell Lines for the Susceptibility and Expression of Pattern Recognition Receptors upon Influenza Virus Infection

**DOI:** 10.3390/v10060312

**Published:** 2018-06-07

**Authors:** Milton Thomas, Max Pierson, Tirth Uprety, Laihua Zhu, Zhiguang Ran, Chithra C. Sreenivasan, Dan Wang, Ben Hause, David H. Francis, Feng Li, Radhey S. Kaushik

**Affiliations:** 1 Department of Biology and Microbiology, South Dakota State University, Brookings, SD 57007, USA; miltontn@gmail.com (M.T.); max.pierson@cardinalhealth.com (M.P.); Tirth.Uprety@sdstate.edu (T.U.); laihuazhu@yahoo.com (L.Z.); zhgran@sina.com (Z.R.); Chithra.Sreenivasan@sdstate.edu (C.C.S.); Dan.Wang@sdstate.edu (D.W.); Feng.Li@sdstate.edu (F.L.); 2 BioSNTR, Brookings, SD 57007, USA; 3 Cambridge Technologies, Oxford Street Worthington, MN 56187, USA; bhause@cambridgetechnologies.com; 4 Department of Veterinary and Biomedical Sciences, South Dakota State University, Brookings, SD 57007, USA; David.Francis@sdstate.edu

**Keywords:** MK1-OSU, SD-PJEC, respiratory epithelial cells, influenza, innate immunity, TLR, RIG-I-like receptor

## Abstract

Influenza viruses infect the epithelial cells of the swine respiratory tract. Cell lines derived from the respiratory tract of pigs could serve as an excellent in vitro model for studying the pathogenesis of influenza viruses. In this study, we examined the replication of influenza viruses in the MK1-OSU cell line, which was clonally derived from pig airway epithelium. MK1-OSU cells expressed both cytokeratin and vimentin proteins and displayed several sugar moieties on the cell membrane. These cells also expressed both Sial2-3Gal and Sial2-6Gal receptors and were susceptible to swine influenza A, but not to human B and C viruses. Interestingly, these cells were also permissive to infection by influenza D virus that utilized 9-*O*-acetylated glycans. To study the differences in the expression of pattern recognition receptors (PRRs) upon influenza virus infection in the respiratory and digestive tract, we compared the protein expression of various PRRs in MK1-OSU cells with that in the SD-PJEC cell line, a clonally derived cell line from the porcine jejunal epithelium. Toll-like receptor 7 (TLR-7) and melanoma differentiation-associated protein 5 (MDA5) receptors showed decreased expression in influenza A infected MK1-OSU cells, while only TLR-7 expression decreased in SD-PJEC cells. Further research is warranted to study the mechanism behind the virus-mediated suppression of these proteins. Overall, this study shows that the porcine respiratory epithelial cell line, MK1-OSU, could serve as an in-vitro model for studying the pathogenesis and innate immune responses to porcine influenza viruses.

## 1. Introduction

Influenza viruses belong to the Orthomyxoviridae family and are classified as types A, B, C, and D based on the antigenic properties of two structural proteins—matrix1 and nucleocapsid (https://www.cdc.gov/flu/about/viruses/types.htm, accessed on 11 February 2018). Pigs are susceptible to A, B, C, and D types of influenza viruses and thus could serve as excellent models for studying pathogenesis and host factors affecting influenza infection. Swine influenza A virus (SIV) infection is an acute respiratory disease endemic to swine populations world-wide. Most of the SIV infections are caused by three subtypes—H1N1, H3N2, and H1N2 [1,2]. The pig airway lining possesses both Sial2-3Gal and Sial2-6Gal receptors [3] and therefore is susceptible to human, avian, and swine influenza A viruses. Co-infection of pigs with human and avian influenza viruses enables the emergence of new strains through gene reassortment. Although the zoonotic potential has not been confirmed, sero-surveillance and virus isolation studies have indicated that swine herds are also susceptible to influenza B [4,5] and C [6,7] infections. Additionally, type D influenza virus was recently discovered in pigs and has been found to be prevalent in swine herds across the United States [8,9,10].

In vitro experiments to study the pathogenesis of influenza viruses were primarily conducted in various mammalian cell lines such as Madin-Darby Canine Kidney (MDCK), baby Hamster Kidney (BHK), A549, and the African Green Monkey Kidney (VERO) cells [11,12,13]. Recently, many studies have focused on using pig models to understand the pathogenesis of SIV. Pigs are the natural host of SIV and possess anatomic and physiologic similarities to humans [14,15]. Thus, in vitro and in vivo swine models could provide a suitable platform to learn about influenza virus biology and pathogenesis. The newborn pig tracheal (NPTr) cell line was established in 2003 and has been used for influenza research [16,17,18]. Other in vitro research was conducted using tracheal explants and lung slices of pigs [19,20,21]. In some other studies, progenitor/stem epithelial cells derived from porcine lungs and progenitor epithelial cells isolated from bone marrow have also been used [22,23].

During influenza infection, the innate immune system detects the presence of virus/viral antigens through the recognition of pathogen-associated molecular patterns (PAMPS) by pattern recognition receptors (PRRs). The PRRs include Toll-like receptors (TLRs) and RIG-I-like receptors (RLRs) that are present in epithelial cells, macrophages, dendritic cells, and natural killer (NK) cells [24]. The recognition of influenza virus by PRRs stimulates the transcription of various cytokines and chemokines such as type I, II, and III Interferons (IFN), and Interleukins (*IL-1*, *IL-6*, and *IL-8*). Epithelial cells produce type I and III IFNs (-α and -β) leading to the downstream activation of interferon-stimulated genes (ISGs), which results in the antiviral response [17,25,26,27,28,29].

Although the intestinal epithelial lining is not the primary target of the influenza virus, recent case reports indicate that Influenza A can successfully replicate in intestinal cells of humans. The 2009 H1N1 pandemic infection caused clinical symptoms such as diarrhea, vomiting, and abdominal pain in the patients [30,31,32]. Additionally, there were incidents where influenza virus was detected in the stool of the patients [33,34]. But, fecal shedding of the virus was not observed in pigs when infected with SIV in our previous experiment [35]. Interestingly, the pig intestinal epithelial lining carries both Sial2-3Gal and Sial2-6Gal receptors for avian, swine, and human influenza viruses [3]. Recently, a new porcine intestinal epithelial cell line was established (SD-PJEC) by subcloning the IPEC-J2 cell line [36]. Various swine influenza A viruses attained high titers but influenza B virus grew poorly in these cells [36]. The comparison between the respiratory and intestinal epithelial cellular systems could provide a better understanding of the differences in the expression of PRRs and the possible reason for the resistance of intestinal cells to SIV infection in vivo. 

Here, we hypothesized that the newly developed porcine airway cell line (MK1-OSU) would express various sugar moieties and would be susceptible to influenza viruses. Further, MK1-OSU and SD-PJEC cell lines would show differential expression of various TLRs, and RLRs in response to influenza infection. To test these hypotheses in this study, we first biochemically characterized porcine airway epithelial cells (MK1-OSU) for the presence of various surface sugar moieties including those important for influenza virus infection and tested their susceptibility to various influenza viruses. We compared the expression of various TLR and RLR proteins between the MK1-OSU and SD-PJEC cell line in response to influenza virus infection. We found that the MK1-OSU cell line possessed an epithelial phenotype, expressed several surface sugar moieties, and was susceptible to influenza virus A and D infection. A variable number of both MK1-OSU and SD-PJEC cells expressed various TLR and RLR proteins. Infection with influenza viruses caused a significant reduction in the expression of TLR-7 and MDA5 in MK1-OSU cells and TLR-7 in SD-PJEC cells at the protein level.

## 2. Materials and Methods 

### 2.1. Cell Lines and Culture Conditions

The MK1-OSU was kindly provided by Dr. Mahesh Khatri (Food Animal Health Research Program, Ohio Agricultural Research and Development Center, The Ohio State University, Wooster, OH, USA). The MK1-OSU is a newly established and spontaneously immortalized cell line derived from the distal trachea and proximal lung tissue of a 5 week old piglet (Khatri et al., unpublished data). Madin-Darby Canine Kidney (MDCK) and MK1-OSU cells were grown in Dulbecco’s modified Eagle’s medium (DMEM; GIBCO BRL Cat. # 11965-092, Invitrogen Corporation, Grand Island, NY, USA) supplemented with 10% fetal bovine serum (FBS; Atlanta Biologicals, Lawrenceville, GA, USA), 100 U/mL Penicillin and 100 µg/mL Streptomycin (Invitrogen, Grand Island, NY, USA), and 2 mM L-glutamine. MK1-OSU cells used in this experiment were between passages 90 and 100. 

The SD-PJEC cell line was derived from the porcine jejunal epithelial IPEC-J2 cell line by sub cloning and it represented a more homogenous cell population [36]. These cells were grown in DMEM: Ham’s F-12 (1:1) medium (Invitrogen, Grand Island, NY, USA) supplemented with 5% FBS, 1% insulin-transferring selenium (ITS) supplement (Invitrogen, Grand Island, NY, USA), 5 ng/mL mouse epidermal growth factor (EGF) (Invitrogen, Grand Island, NY, USA), 100 U/mL Penicillin, and 100 µg/mL Streptomycin. All cell cultures were maintained in a humidified incubator at 37 °C with 5% CO_2_.

### 2.2. Phenotyping of MK1-OSU Cells by Immunohistochemistry 

The MK1-OSU cells were stained with antibodies which recognized various epithelial, fibroblast, and smooth muscle markers using the protocol as described previously [37,38]. Briefly, MK1-OSU cell cultures were harvested from T-25 or T-75 tissue culture flasks and washed with PBS. Cell number was adjusted to 10^6^/mL and 100 µL of cell suspension was used for preparing cytospins using a cytofuge (Cytospin 3; Thermo Shandon Ltd., Cheshire, UK). The cytospins were air-dried for 2 h, fixed in acetone for 7 min, and stored at 4 °C until later use. For antibody staining, slides were equilibrated at room temperature, rehydrated in PBS, and then blocked for non-specific protein binding with PBS containing 1% goat serum. After washing slides three times with PBS, cells were incubated in PBS containing 0.3% hydrogen peroxide and 0.01% Sodium azide to block endogenous peroxidase activity. The presence of cytokeratin, vimentin, α-smooth muscle actin (ASMA), and desmin proteins was detected by immunohistochemical (IHC) staining using anti-cytokeratin mAb C6909 (IgG2a isotype), anti-vimentin mAb V5255 (IgM isotype), anti-ASMA mAb A2547 (IgG2a isotype), and anti-desmin mAb D1033 (IgG1). Monoclonal antibodies M9144 (IgG2a isotype), M9269 (IgG1 isotype), and M5170 (IgM isotype) were used as isotype-matched controls. Also, a negative control without primary antibody staining was also used. Cytospins were incubated with 100 µL of primary antibodies (Sigma-Aldrich, St. Louis, MO, USA) for 1 h at a 1 μg/mL concentration. Slides were washed three times with PBS and then incubated with 100 µL/slide of isotype-specific, biotinylated goat anti-mouse IgG2a, IgG1, or IgM antisera (1:2000 dilution; Caltag Laboratories) for 30 min. Antibody labeling was visualized by incubating with HRP–streptavidin solution for 30 min followed by the addition of Ready-to-use (RTU) diaminobenzene (DAB) substrate (Vector Laboratories, Burlingame, CA, USA). Cytospins were counterstained with hematoxylin and examined under the light microscope. Images were taken at 20× magnification using an Olympus AX70 microscope.

### 2.3. Lectin Binding Assay

The expression of a panel of 21 lectins was determined according to the protocol described by the manufacturer (Vector Laboratories, Burlingame, CA, USA). Briefly, 0.5 × 10^6^ cells of MK1-OSU cells were harvested and transferred to a U-bottom 96 well assay plate. Cells were then treated with biotinylated lectins at a 1 µg/well concentration for 1 h. Cells were washed three times with PBS and then incubated for 30 min with streptavidin-FITC (1:200 dilution). Negative controls for each lectin included cells stained without any biotinylated lectin but incubated with streptavidin-FITC treatment. The lectin-binding profile of cells was measured using a FACSCalibur cytometer (Becton Dickinson, San Jose, CA, USA). The experiment was repeated three times using MK1-OSU cells. The specificity of lectin binding was validated by incubating the cells with lectins treated with specific carbohydrate ligands/inhibitors to block the lectin binding to cell surface sugar moieties. Details of the lectins, inhibitors, and specificity of the lectins binding to various sugars are listed in Table 1.

The expression of Sial2-3Gal and Sial2-6Gal receptors on MK1-OSU cells was also quantified using the above protocol and compared to MDCK cells using a lectin binding assay. *Maackia amurensis* agglutinin/lectin-2 (MAA/MAL-II) is specific for Sial2-3Gal and *Sambucus nigra* agglutinin (SNA) (Vector Laboratories, Burlingame, CA, USA) is specific for Sial2-6Gal receptors.

### 2.4. Culture of Swine and Human Influenza Virus and Infection of Cells

Seven influenza viruses were used in this study. These include—

(1) A/California/04/2009 (pdm09/CA04); (2) A/swine/Minnesota/2073/2008(H1N1-MN08); (3) A/swine/Iowa/0855/2007(H3N2-IA07); (4) B/Florida/4/2006 (FL06); (5) B/Brisbane/60/2008 (BR08); (6) C/Victoria/2/2012 (ICV); and (7) D/Swine/Oklahoma/1334/2011 (DOK) [8]. All the viruses were propagated in MDCK cells at 37 °C, except for ICV, which was grown at 33 °C and were stored at −80 °C. Briefly, MDCK cells grown in T75 flasks were treated with 100 μL of virus suspension added to 1.9 mL DMEM supplemented with 0.3% bovine serum albumin (BSA, Sigma-Aldrich, St. Louis, MO, USA). After 1 h incubation either at 37 or 33 °C for adsorption, cells were washed with PBS. The DMEM media containing 0.3% BSA and tolylsulfonyl phenylalanyl chloromethyl ketone-treated (TPCK) trypsin at the concentration of 1 µg/mL (Thermo Scientific Pierce, Rockford, IL, USA) were added. Cells were monitored daily for cytopathic effects and viruses were harvested when 80% of cells were detached from the flask and the supernatant was stored at −80 °C. The viruses were titrated in MDCK cells by preparing serial ten-fold dilutions. Virus titers were calculated by using the Reed and Muench method [39]. 

For further experiments, 0.5 × 10^6^ cells of various cell types were seeded in a six-well plate and incubated at 37 °C. After 18 h, cells were infected with the virus at MOI of 0.01 and incubated for 1 h at 37 °C. The virus suspension was then removed, cells were washed with PBS, and 2 mL of DMEM media supplemented with 1 µg of TPCK trypsin/mL and 0.3% BSA was added and incubated for the length of the experiment at 37 °C.

### 2.5. Indirect Immunoflourescence Assay for Virus Detection

Infection of MK1-OSU cells with influenza A (MN08, IA07) viruses was detected using an indirect immunoflourescence assay. Briefly, cells were infected as described above and media was removed at 24 h post-infection and cells were fixed with 200 µL of 2% paraformaldehyde in PBS. Cells were permeabilized and blocked for non-specific binding of proteins using 1 mL of 0.1% Triton-X and 2% BSA in PBS. The fixed cells were then incubated with primary antibodies—mouse anti-influenza A nucleoprotein (AbD Serotec, Raleigh, NC, USA)—for 1 h at a concentration of 1 µg/well. After washing with PBS, cells were treated with goat anti-mouse IgG-Alexa 488 (Invitrogen, Grand Island, NY, USA) secondary antibody for 1 h. Cells were washed with PBS and examined under an inverted Olympus AX70 fluorescent microscope at 20× magnification.

### 2.6. Determination of Percentage of MK1-OSU Cells Infected Using Flow Cytometry

MK1-OSU cells were infected using MN08 or IA07 viruses. After 24 h, the percentage of infected cells and mean fluorescence were determined using flow cytometry. Non-infected cells were used as a control. Cells were fixed and permeabilized using BD Cytofix/Cytoperm™ (BD Biosciences, San Jose, CA, USA). After blocking with 1% goat serum, cells were incubated with primary antibodies against the nucleoprotein of influenza A virus (AbD Serotec, Raleigh, NC, USA) for 1 h. Cells were stained using goat anti-mouse IgG-Alexa 488 (Invitrogen, Grand Island, NY, USA). Percentage of cells and mean fluorescence intensity (MFI) were measured using a FACSCalibur cytometer (Becton Dickinson, San Jose, CA, USA). The gating for MFI was set at 2 for the unlabeled cells and the data for labelled cells was normalized to the unlabeled cells as described previously [40]. 

### 2.7. Growth Kinetics of Influenza Viruses in MK1-OSU, SD-PJEC and MDCK Cells

The growth kinetics of five influenza viruses were measured in MK1-OSU, SD-PJEC, and MDCK cells. Cells were infected as described above at 0.01 MOI and 100 µL of media was sampled out at 12 h intervals until 72 h. Virus titers, defined as the log_10_ 50% tissue culture infective dose (TCID50), were determined at various time points according to the protocol described elsewhere [41].

### 2.8. Estimation of Pattern Recognition Receptors (PRRs) Using Flow Cytometry

Basal levels and alterations in the protein level expression of PRRs in MK1-OSU and SD-PJEC cells following SIV (MN08 and IA07) infection were determined by flow cytometry. We measured expressions of TLRs-2, -3, -4, -5, -6, -7, -8, -9, RIG-I, and MDA5 at 24 h and TLR 7, RIG-I, and MDA5 at 6 h after infection for both cell types After 6 or 24 h, media was removed, and cells were harvested and transferred to a 96-well assay plate. Cells were then fixed and permeabilized following the manufacturer’s protocol for BD Cytofix/Cytoperm™ (BD Biosciences, San Jose, CA, USA). The fixed cells were blocked for non-specific binding of primary antibodies using 1% goat serum. Cells were then incubated with 50 µL of primary antibodies (Table 2) for 1 h at a concentration of 1 µg/well. After washing, cells were treated with 1:2000 diluted biotinylated secondary antibody for 30 min. Cells were further incubated for 30 min with streptavidin-FITC (1:200 dilution) for staining. All the incubations were performed at 4 °C. Negative controls were cells without first and secondary antibody treatment. Finally, PRRs expression on the cells was measured using a FACSCalibur cytometer (Becton Dickinson, San Jose, CA, USA).

### 2.9. Real-Time RT-PCR Assays for Pattern-Recognition Receptors (PRRs)

The effect of influenza virus infection on the gene expression of PRRs in MK1-OSU and SD-PJEC cells was measured using real-time quantitative RT-PCR. Cells were infected with MN08 or IA07 influenza viruses at 0.01 MOI and incubated for 24 h. Control cells without infection were also maintained for the same time periods. After incubation, total RNA was extracted from each well using the RNeasy mini kit (Qiagen, Valencia, CA, USA) and was quantified using an ND-1000 UV-visible light spectrophotometer (NanoDrop Technologies, LLC, Wilmington, DE, USA). For each sample, 1 µg of RNA was reverse-transcribed to 50 µL of cDNA using TaqMan Reverse transcriptase reagents (Applied Biosystems, NJ, USA) and stored at −20 °C. All the experiments were repeated three times.

Details of oligonucleotides used for real-time RT-PCR in this study are given in Table 3. The reaction mix (25 µL final volume) consisted of 12.5 µL of SYBR green/ROX PCR master mix (SABiosciences, Frederick, MD, USA), 0.5 to 1.0 µL of each primer (200–400 nM), 2 µL of the cDNA preparation, and a variable amount of nuclease-free water. The RT-PCR conditions used were 95 °C for 10 s, annealing temperatures ranging from 55–60 °C for 15 s, and an extension temperature of 72 °C for 20 s for 40 cycles. The relative expression of genes was calculated using the ΔCt (Ct of the target gene—Ct of the housekeeping gene) method. RPL4 was used as the housekeeping gene to calculate the change in expression of the genes. The specificity of each RT-PCR product was confirmed by analyzing the melting curves.

### 2.10. Statistical Analysis

The Student’s *t*-test was used to compare the MAL-II and SNA lectin binding and the percentage of virus infection. The expression of PRRs on the cells and quantity of mRNA were statistically compared using a non-parametric multiple comparison test in GraphPad Prism v7.03. Statistical difference was defined at *p* < 0.05.

## 3. Results

### 3.1. MK1-OSU Cells are Phenotypically Epithelial Cells

**Figure 1:**
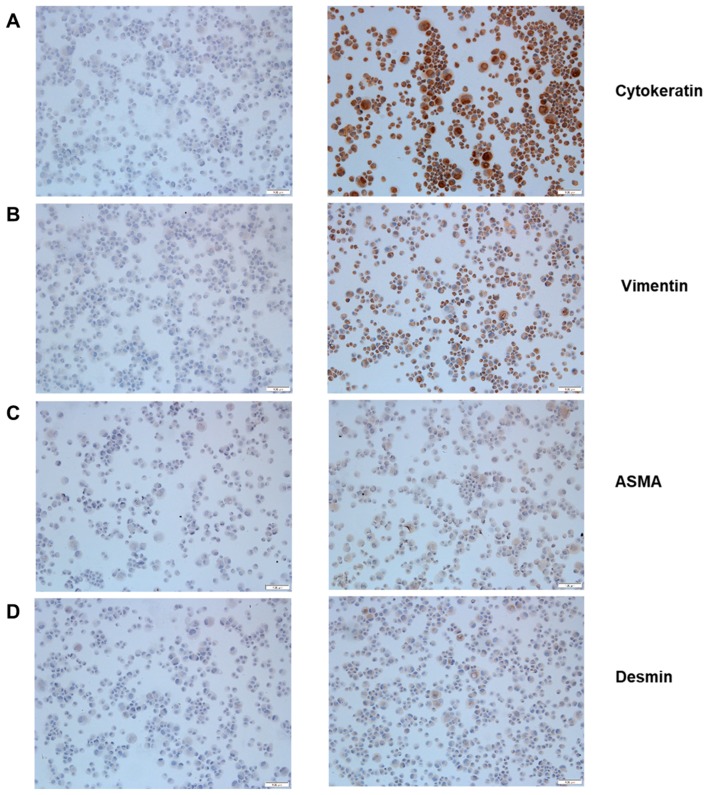
Immunohistochemical staining of MK1-OSU cells for phenotypic markers: cytokeratin, vimentin, α-smooth muscle actin (ASMA), and desmin. Cytospins were prepared and stained with marker-specific monoclonal antibodies. Isotype controls for antibodies were stained concurrently. Cells were stained with cytokeratin (**A**), vimentin (**B**), ASMA (**C**), and desmin (**D**) specific monoclonal antibodies and their isotype controls. The left panel represents the isotype controls for cytokeratin, vimentin, ASMA, and desmin, respectively. As shown in the right panel, cells stained positively for epithelial cell marker, cytokeratin, and fibroblast marker vimentin. The images are representative of three independent experiments. The scale bars on the bottom left corner of each image represents 100 µm length.

The MK1-OSU cells were clonally derived from the airway epithelial cells of a 5-week-old piglet. Cells were stained for cytokeratin, vimentin, α-smooth muscle actin (ASMA), and desmin to determine whether these cells are phenotypically epithelial in nature. These four proteins are markers for epithelial cells, fibroblast, smooth muscle, and smooth and striated muscles, respectively. As shown in Figure 1, cells positively stained for cytokeratin and vimentin but were either negative or faintly stained for ASMA and desmin, suggesting that MK1-OSU cells possess an epithelial phenotype.

### 3.2. Lectin Binding Profile of MK1-OSU Cells Indicated the Presence of Heterogeneous Cell Surface Sugar Moieties

**Figure 2:**
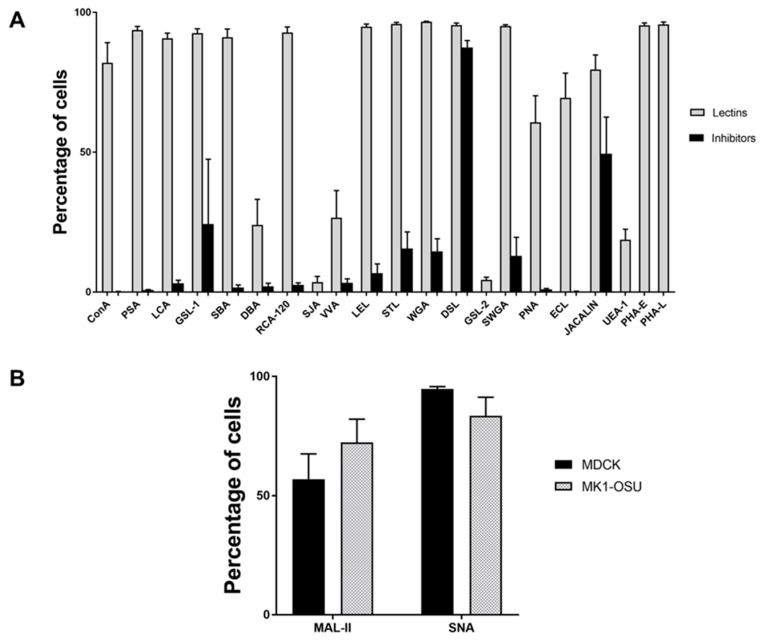
Lectin binding profile of MK1-OSU cells. (**A**) MK1-OSU cells were incubated with biotinylated lectins and were stained using streptavidin-FITC. Samples were analyzed using a FACSCalibur flow cytometer. Values represent the average for three to five independent experiments ± SE. (**B**) Differential expression of sialic acid receptors for influenza A virus in MK1-OSU and MDCK cells. Both MK1-OSU and MDCK cells were incubated with biotinylated lectins binding to Sial2-3Gal (MAL-II) and Sial2-6Gal (SNA) and were stained using streptavidin-FITC. Samples were analyzed using a FACSCalibur flow cytometer. Values represent the average for four experiments ± SE.

The presence of sugar moiety and percentage of MK1-OSU cells binding to various lectins were analyzed using flow cytometry. A panel of 21 lectins and corresponding inhibitors (Table 1) were used for this experiment. The percentages of cells binding to various lectins varied broadly, indicating the presence of glycans/various sugars in differential quantities on the MK1-OSU cells (Figure 2A). The highest binding percentages (>90%) were observed for lectins SBA, WGA, RCA-120, GSL-1, PSA, LCA, SWGA, DSL, LEL, STL, PHA-E, and PHA-L. Most inhibitors significantly blocked/reduced the lectin binding, confirming the specific binding of the lectin to MK1-OSU cells. However, DSL binding was not significantly inhibited by the treatment with sugar, indicating that the binding could be either non-specific or that a low concentration of inhibitor was used to block its binding. CON-A (81.98 ± 7.18%) and Jacalin (79.54 ± 5.21%) also showed a high percentage of binding to MK1-OSU cells. Similar to DSL, Jacalin was only inhibited moderately by the addition of inhibitory sugar. Lectins PNA and ECL exhibited moderate binding (60.69 ± 9.49 and 69.43 ± 8.80% respectively). Lectins DBA (23.98 ± 9.13%), VVA (26.56 ± 9.69%), and UEA-1 (18.65 ± 3.79%) showed only low-level binding to the cells. Two lectins, SJA and GSL-2, were bound to only <5% cells and therefore no inhibitors were used to check the specificity of these lectins. If all the cells possessed or lacked the same receptors, the binding affinity of lectins would be uniform and either 100% or 0% of cells would show binding to lectins. However, the binding percentages for various lectins were between 0 and 100% for MK1-OSU cells, indicating that the distribution of some sugar moieties is not uniform across the cells. The lectin binding pattern observed in this study is suggestive of the presence of heterogeneous subpopulations within the MK1-OSU cell line and the majority of tested lectins showed significant binding to these cells.

### 3.3. MK1-OSU Cells Expressed Sial2-3Gal and Sial2-6Gal Receptors

The expression of Sial2-3Gal and Sial2-6Gal receptors in pig tracheal epithelial cells has been shown in previous studies [3,42]. This property renders pig susceptible to swine, avian, and human viruses. Both MK1-OSU and MDCK cells were incubated with biotinylated MAL-II specific for Sial2-3Gal and SNA specific for Sial2-6Gal and stained using streptavidin-FITC. The expression of receptors was measured using flow cytometry. As shown in Figure 2B, both MK1-OSU and MDCK cell types had a similar level of expression for Sial2-3Gal (72.28% ± 9.76 and 56.86% ± 12.26; *p* = 0.07) and Sial2-6Gal (83.46% ± 7.78 and 94.70%± 0.97; *p* = 0.20) receptors. These results suggested that MK1-OSU cells could be susceptible to swine, avian, and human influenza viruses.

### 3.4. MK1-OSU Cells Were Susceptible to SIV Infection 

**Figure 3:**
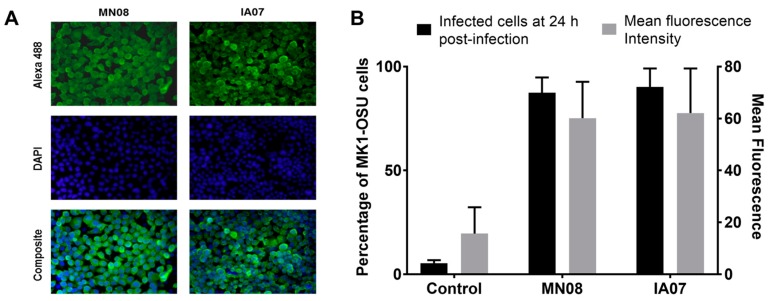
MK1-OSU cells infected with influenza A (MN08 and IA07) virus. (**A**) Cells were infected with MN08 or IA07 viruses at MOI of 0.01 and incubated for 24 h at 37 °C and were fixed with 2% paraformaldehyde in PBS. The fixed cells were incubated with mouse anti-influenza A nucleoprotein and were stained with goat anti-mouse IgG-Alexa 488 (green color) for 1 h. Nuclei were stained using DAPI (blue color) and cells were washed with PBS and examined under an inverted Olympus AX70 fluorescent microscope at 20× magnification. (**B**) Percentage of MK1-OSU cells infected with influenza A (MN08 and IA07) virus. MK1-OSU were infected at MOI of 0.01 and incubated for 24 h at 37 °C. Following incubation, cells were fixed and permeabilized using BD Cytofix/Cytoperm™. After blocking with 1% goat serum, cells were incubated with primary antibodies against the nucleoprotein of influenza A virus and were stained using goat anti-mouse IgG-Alexa 488. Percentage of cells and mean fluorescence intensity were measured using a FACSCalibur cytometer. Values represent the average for three experiments ± SE.

The susceptibility of MK1-OSU cells to influenza infection was determined by infecting these cells with influenza A (MN08 and IA07) viruses. Cells were stained for the NP protein at 24 h post-infection using IFA and viewed under a fluorescent microscope at 20× magnification (Figure 3A). The results indicated that MK1-OSU cells were susceptible to both subtypes (H1N1 and H3N2) of SIV Similar to IFA, cells were also stained using antibodies against the NP protein that were conjugated to Alexa 488 and analyzed by Flow cytometry. The mean percentage of cells infected at 24 h post-infection was 87.42 and 90.23 for MN08 and IA07, respectively. Normalized mean fluorescence intensity was also higher for infected cells compared with control non-infected cells (Figure 3B). These results supported the findings obtained by IFA, which indicated that MK1-OSU cells were susceptible to SIV.

**Figure 4:**
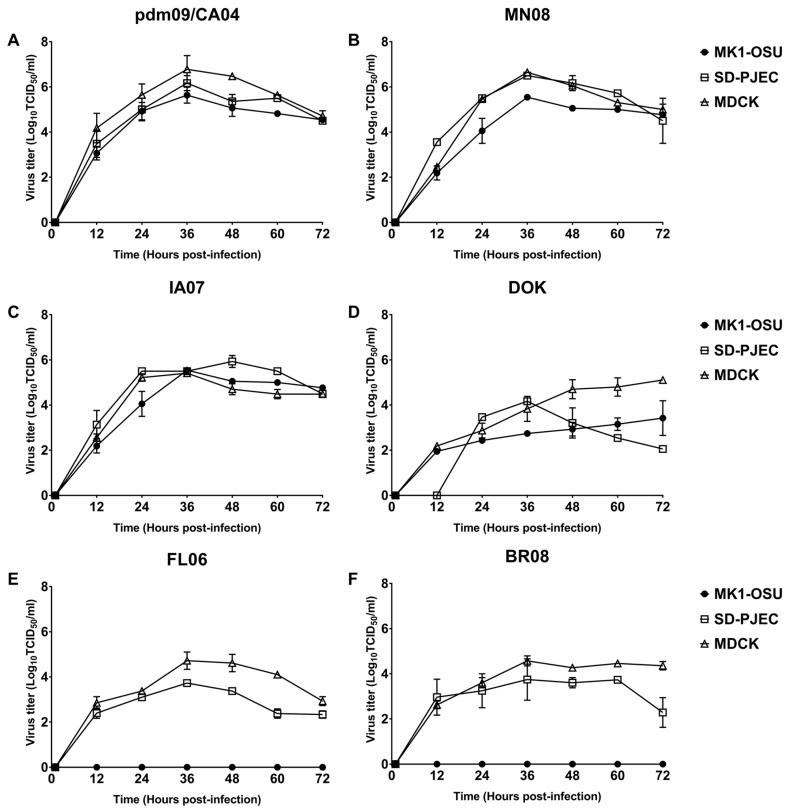
Growth kinetics of type A and D influenza viruses in MK1-OSU, SD-PJEC, and MDCK cells. Growth kinetics of type A and D influenza viruses during 72 h post-infection were measured in MK1-OSU, SD-PJEC, and MDCK cells. Briefly, cells were infected with the influenza viruses at MOI of 0.01 and 100 µL of media was sampled at 12 h intervals until 72 h. Viral 50% infective doses were expressed as log_10_ TCID_50_/mL. Values represent the mean ± SE of three experiments for (**A**) pdm09/CA04, (**B**) MN08, (**C**) IA07, (**D**) DOK, (**E**) FL06, and (**F**) BR08.

Viral replication kinetics of all the four types of influenza viruses were performed on MK1-OSU and SD-PJEC cells and were compared to MDCK cells at 37 °C for 72 h (Figure 4). Influenza A and D viruses replicated to titers comparable to MDCK cells (Figure 4A–D). Similar to MDCK cells, MK1-OSU and SD-PJEC cells demonstrated a peak titer at 36 h post-infection for influenza A Pdm09/CA04 and MN08. Interestingly, SD-PJEC had a peak titer at 48 h post-infection compared to MDCK cells when infected with IA07. MK1-OSU cells also supported the productive replication of the swine isolate of influenza D (DOK) virus. DOK usually attains a peak titer at five days post-infection and this could be the reason for the low titers exhibited by the DOK in MK1-OSU and MDCK cells when compared to influenza A viruses (Figure 4D). However, in SD-PJEC cells, the DOK attained a peak titer of 4.17 log_10_ TCID_50_/mL at 36 h and declined thereof, indicating that these cells poorly supported the growth of DOK. The influenza B viruses did not replicate in MK1-OSU cells to yield appreciable titers but attained lower titers in SD-PJEC cells compared to MDCK cells. The peak titers in SD-PJEC and MDCK cells were 3.72 ± 0.003 and 4.72 ± 0.39 for FL06 and 3.75 ± 0.92 and 4.57 ± 0.22 for BR08, respectively (Figure 4E,F). The ICV did not grow in all the cell lines at 37 °C.

### 3.5. Influenza Infection Differentially Affected the Expression of TLRs and RLRs in MK1-OSU and SD-PJEC Cells at 24 h 

**Figure 5:**
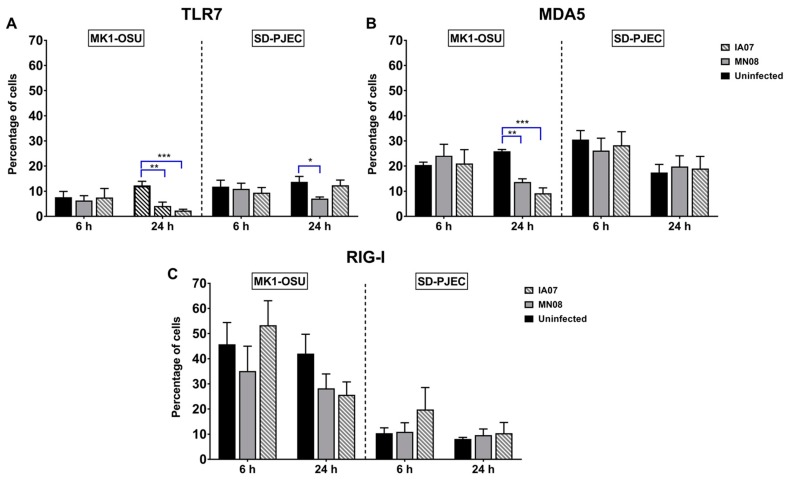
Expressions of TLRs and RLRs in MK1-OSU and SD-PJEC cells at 6 h and 24 h post-infection determined using a flow cytometer. MK1-OSU and SD-PJEC cells were infected with MN08 and IA07 viruses at MOI of 0.01 and incubated for 6 and 24 h at 37 °C. Cells were fixed and permeabilized using BD Cytofix/Cytoperm, incubated with primary antibodies, treated with biotinylated secondary antibody, and stained with streptavidin-FITC. (**A**) TLR-7 expression was decreased by both MN08 and IA07 in MK1-OSU cells 24 h post-infection but only by MN08 in SD-PJEC cells. (**B**) Expressions of MDA5 were reduced by MN08 and IA07 infection in MK1-OSU cells at 24 h but did not affect the expressions in SD-PJEC cells. (**C**) RIG-I did not alter due to treatments in both cell lines. Bars represent the mean for four to six experiments ± SE *p* < 0.05 (*) represented a significant difference between uninfected and infected cells. ** and *** represents *p* < 0.005 and *p* < 0.0005 respectively.

After determining the susceptibility of MK1-OSU cells to influenza infection, we investigated the expression of various TLRs and RLRs before and after influenza virus infection in these cells. Furthermore, the presence and expression levels of TLRs and RLRs were investigated in SD-PJEC cells and compared with MK1-OSU cells. Both MK1-OSU and SD-PJEC cells expressed TLRs-2, -4, -9, RIG-I, and MDA5 receptors; however, the percentage of receptor-positive cells varied between these two cell types for TLR-6, TLR-8, and RIG-I (Figure 5 and Table 4). Alterations in the protein expressions of various TLRs and RLRs (RIG-I and MDA5) following infection with SIV were evaluated at 6 h (Figure 5) and 24 h (Figure 5 and Table 4) post-infection using flow cytometry. In MK1-OSU cells, MN08 and IA07 significantly reduced the expressions of TLR-7 and MDA5 24 h post-infection compared to control non-infected cells. These PRRs are important for influenza virus detection and decreased levels of these receptors could possibly help in the virus propagation. Conversely, levels of RIG-I and MDA5 were not altered by infection in SD-PJEC cells. Interestingly, TLR-7 expression was only reduced by MN08 in SD-PJEC cells and not by IA07, suggesting that there could be strain-dependent variation in pathogenesis within influenza A viruses. Percentage of cells expressing proteins TLRs-2, -4, -5, -6, -8, and -9 did not alter during the infection for both cell lines at 24 h post-infection (Table 4). Expressions of TLR-7, RIG-I, and MDA5 were also analyzed 6 h post-infection, but were found to be similar for infected and uninfected cells in both cell lines (Figure 5). TLR-3 was not detected in either cell types, possibly due to a lack of cross-reactivity of detection antibodies with swine antigens. 

**Figure 6:**
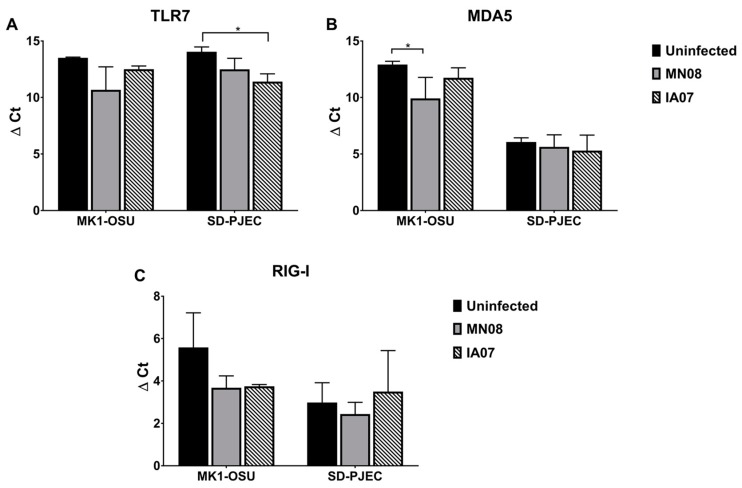
Gene expressions of TLRs and RLRs in MK1-OSU and SD-PJEC cells 24 h post-infection determined using real-time RT_PCR. MK1-OSU cells were infected with MN08 or IA07 viruses at MOI of 0.01 and incubated for 24 h at 37 °C. Values represent the mean ± SE of ΔCt (Ct of target—Ct of housekeeping gene) for three experiments. The difference in the Ct values has an inverse relationship with the gene expression. (**A**) *TLR-7* mRNA increased at 24 h in SD-PJEC cells infected with IA07. (**B**) *MDA5* gene expression increased in MK1-OSU cells infected with MN08 at 24 h. (**C**) No difference in the *RIG-I* mRNA level due to SIV infection in both cell types. *p* < 0.05 (*) represented a significant difference between uninfected and infected cells.

We further investigated whether the changes in the protein levels of TLRs and PRRs in MK1-OSU and SD-PJEC cells were reflected at the transcript level using real-time RT-PCR. The MK1-OSU and SD-PJEC cells were infected with MN08 or IA07 viruses at MOI of 0.01 and incubated for 24 h at 37 °C. The mRNA expression levels of *TLR-7*, *MDA5*, and *RIG-I* were determined by real-time RT-PCR. The mean ΔCt (Ct of the target gene—Ct of the housekeeping gene) values, which represent the change in gene expression of the target gene normalized to a housekeeping gene, are given in Figure 6. The difference in the Ct values has an inverse relationship with the gene expression. The results indicated that *TLR-7* mRNA increased at 24 h in SD-PJEC cells infected with IA07 while MN08 infection did not alter the gene expression. In MK1-OSU cells, the levels of *TLR-7* mRNA for uninfected cells and infected cells were similar. *RIG-I* mRNA was not affected by SIV infection in both cell types. Further, *MDA5* gene expression was elevated in MK1-OSU cells infected with MN08 compared to uninfected cells while there were no alterations at the gene level in SD-PJEC cells following SIV infection. These findings for *TLR-7* and *MDA5* mRNA concentrations did not correlate with the protein expressions observed in this study. Moreover, the increase in mRNA levels of *MDA5* and subsequent decrease in the protein level could be suggestive of a post-transcriptional inhibitory mechanism by SIV. 

## 4. Discussion

Epithelial cells lining the respiratory tract of pigs are the cells that are targeted first by the SIV. Conversely, these cells could also act as the first line of defense against viral infections. Therefore, the porcine respiratory epithelial cells could serve as an important in vitro model for studying viral pathogenesis and innate immune responses to SIV. Moreover, research using primary cultures and explants of swine respiratory epithelial cells has gained greater focus after the infections caused by the 2009 pandemic H1N1 [31] and 2011 H3N2 variant [43] viruses that were of swine origin. However, the NPTr cell line, which is a non-transformed continuous cell line derived from 2 day old piglet, is the sole known cell line established from pig tracheal epithelium [18]. The MK1-OSU cell line used in this study is relevant because it provides an additional platform to study the pathogenesis of respiratory diseases. Further, it was derived from a 5 week old pig and the respiratory system at that age is functionally similar to that of an adult pig [44]. 

A high percentage of the MK1-OSU cells (>90%) were stained for cytokeratin, suggestive of the epithelial phenotype of these cells. Interestingly, a high proportion of MK1-OSU cells also stained for vimentin, which is a fibroblast cell marker. Previous reports have suggested that epithelial cells, when cultured in vitro, could also stain for vimentin [37,45,46,47,48]. The expression of vimentin in alveolar epithelial cells is necessary for cell migration, wound healing, and lung remodeling [49]. Vimentin is also a marker for epithelial to mesenchymal transition and has a significant role in tumor metastasis. An increased expression of vimentin was observed in malignant tumor cells, which were characterized by chromosomal aberrations [50,51]. Many of the continuous epithelial cell lines, including NPTr, have chromosomal aberrations that are responsible for the indefinite propagation of those cells. It is plausible that vimentin might have a significant role in the propagation of the MK1-OSU cell line, which is a primary but continuous cell line that had been passaged for more than hundred times.

The MK1-OSU cells were further characterized for the presence of various cell surface sugars detected by a panel of 21 lectins. Lectins are proteins that can specifically bind to sugar molecules present on the cell surface or on glycoprotein receptors present on the epithelial or other cells. Lectins possess a high specificity in binding to the sugar ligands and play a significant role in the adhesion of various bacteria and viruses to the epithelium. Thus, the lectin-binding profile of a particular cell type may provide important clues about the possible infectivity of those cells to various bacteria and viruses. Lectins are also currently being explored for developing alternate treatment strategies against infectious diseases because of their ability to inhibit the binding of pathogens to the epithelium and other types of cells [52]. The presence of various lectin-binding receptors in swine airway epithelium has been reported previously [53]. The porcine airway tract epithelium was shown to be rich in *N*-acetyl galactosamine, *N*-acetyl glucosamine, and fucose residues. The results based on the lectin binding profile indicated that MK1-OSU cells expressed galactose, glucose, mannose, fucose, *N*-acetyl glucosamine, *N*-acetyl galactosamine, and many oligosaccharides on their surface. Thus, these cells are rich in a variety of lectin-binding receptors and could be utilized for studying the pathogenesis of infectious diseases that affect the respiratory tract. In addition to the 21 lectins, we also analyzed the presence of Sial2-3Gal and Sial2-6Gal receptors that are specific for influenza virus infectivity using MAL-II and SNA lectins, respectively.

Similar to MDCK cells, a large percentage of MK1-OSU cells expressed both Sial2-3Gal and Sial2-6Gal receptors, indicating the possible infectivity of these cells to influenza viruses. This resembles the pig respiratory tract epithelium where both receptors are abundantly present. The MK1-OSU cells were found to be susceptible to infection with the influenza A strains used in this experiment and could be utilized for studying the pathogenesis of various strains of human and swine influenza A viruses. These findings are also consistent with an earlier study, where MK1-OSU cells were shown to be susceptible to six different strains of influenza A viruses [54]. In this study, we also used influenza B and C viruses for infecting MK1-OSU cells; the BR08 virus that belongs to the Victoria lineage, the FL06 virus that belongs to the Yamagata lineage, and human ICV. Although influenza B and C are human pathogens, studies have indicated that pigs could be susceptible to these viruses [4,5,7]. However, the MK1-OSU cells did not support the growth of these viruses. Additionally, MK1-OSU cells were also infected by recently discovered influenza D/OK virus [8]. Overall, our findings demonstrated the utility of the MK1-OSU cell line for studying the pathogenesis of influenza A and D viruses. 

The second objective of this study was to determine the constitutive expression of various TLRs and RLRs on MK1-OSU and SD-PJEC cells when infected with influenza viruses. A comparison of the level of TLRs and RLRs expression between MK1-OSU and SD-PJEC cells could provide us with a better understanding of the differences in influenza pathogenesis occurring in the respiratory and intestinal tract. We first identified various human TLR and RLR specific antibodies which cross-react with porcine TLRs and RLRs. This was a great step forward as only a limited number of porcine specific TLR and RLR antibodies have been described previously. In this study, both MK1-OSU and SD-PJEC cells constitutively expressed variable levels of TLRs and RLRs. 

The immune response to virus infection is initiated when the host cells recognize viral nucleic acids, either DNA or RNA, through TLRs and RLRs [17,26,27]. TLR-3 and TLR-7/8 are endosomal RNA sensors and recognize dsRNA and ssRNA, respectively. The RLRs, RIG-I and MDA5, are present in the cellular cytoplasm and can detect viral nucleic acid. Recognition of foreign RNA triggers a cascade of signaling that leads to the production of cytokines and chemokines, especially the antiviral interferons. Among RLRs, RIG-I plays a significant role in mammalian cells for influenza virus recognition [55]. In contrast, chickens and ducks have MDA5 as the major sensor for the influenza genome [56,57]. During influenza infection, the expressions of TLR-7, RIG-I, and MDA5 were increased in both in vivo and in vitro studies [55,58]. The findings of the present study were in contrast to previous findings. The percentage of cells expressing TLR-7 and MDA5 was significantly reduced in the MK1-OSU cells when infected with MN08 and IA07. Similarly, the percentage of SD-PJEC cells expressing TLR-7 was also lowered after infection with MN08, but not by IA07. Additionally, the percentage of cells expressing RIG-I did not alter the following SIV infection. These dissimilarities from previous findings could possibly be because of the differences in the characteristics of cells or virus strains used in this study. However, the mRNA levels of *TLR-7* and *MDA5* elevated following the SIV infection in SD-PJEC and MK1-OSU cells, respectively, and these results were similar to previous observations [59,60]. 

Another possibility is that influenza A virus suppressed TLR-7 and MDA5 expressions as a mechanism to evade the host immune system. However, previous research does not demonstrate a direct suppressive effect on these PRRs by the influenza virus. Instead, influenza viruses adopt evasive mechanisms such as NS1 mediated inhibition of the RIG-I signaling cascade, leading to lowered IFN production, the deregulation of general host cell gene-expression, and the elevated expression of genes that suppress cytokine production [61,62,63,64,65,66]. On the other hand, RNA viruses such as vesicular stomatitis virus, encephalomyocarditis virus, and human immunodeficiency virus have devised various mechanisms to degrade the RIG-I protein [67,68,69]. Further research is required to determine the mechanism behind the SIV-mediated direct suppression of TLR-7 and MDA5 as observed in MK1-OSU cells.

A limitation of this study was that primary antibodies against human PRRs were used against proteins of porcine origin. The alignment of complete amino acid sequences for TLR-7 and MDA5 revealed 85% identity between human and swine. The information regarding the percentage identity of the peptide sequences used for generating these antibodies is not available. The percentage identity between the sequences of human and swine PRRs could have influenced the degree of cross-reactivity of antibodies between the species and could be a plausible reason for the expression of these PRRs by a lower percentage of cells.

Our results suggested differential PRRs expression for the two cell lines when infected with SIV. The major difference in the innate immune response between MK1-OSU cells and SD-PJEC cells was that MDA5 expression was not lowered in SD-PJEC cells in spite of having a similar percentage of cells expressing MDA5 in the uninfected condition. It is possible that the intestinal epithelial lining of pigs behaves in a similar manner to SD-PJEC cells, where MDA5-mediated immunity could be playing a predominant role in preventing SIV infection. Furthermore, recent research has underscored the importance of MDA5 in the defense against influenza virus [70]. Attenuated NS1-mutant influenza virus that possessed siRNA against the MDA5 gene was found to be the dominant strain among the viral population in vitro suggesting that evasion of MDA5-mediated immune response is a determinant of influenza proliferation [70]. 

## 5. Conclusions

Overall, this research showed that the MK1-OSU cells are susceptible to various influenza viruses and could be used as an in vitro model for studying the pathogenesis of influenza A and D infections. It provides a novel platform of swine-origin cells for influenza-related studies. This study also demonstrated that SIV suppresses the expression of TLRs and RLRs in the epithelial cells of the respiratory tract; possibly a mechanism to evade detection by the host immune system. Further research needs to be conducted for elucidating the viral strategies responsible for the inhibition of these host cellular proteins.

## Figures and Tables

**Table 1:** Lectins and inhibitors used for determining the lectin binding profile of MK1-OSU cells.

Lectins	Concentration (µg/mL)	Inhibitor	Concentration (mM)
**1. Glucose/mannose group**			
Concanavalin-A (ConA)	10	α-methyl mannoside, α-methyl glucoside	200 each
Pisum Sativum agglutinin (PSA)	20	α-methyl mannoside, α-methyl glucoside	200 each
Lens culinaris agglutinin (LCA)	20	α-methyl mannoside, α-methyl glucoside	200 each
**2. *N*-acetylgalactosamine group**			
Griffonia simplicifolia lectin I (GSL-1)	10	Galactose	400
Soybean agglutinin (SBA)	10	*N*-acetyl galactosamine	200
Dolichohs Biflorus agglutinin (DBA)	10	*N*-acetyl galactosamine	200
Ricinus communis agglutinin (RCA-120)	10	Galactose	200
Sophora japonica agglutinin (SJA)	20	No inhibitor used	
Vicia villosa agglutinin (VVA)	20	*N*-acetyl galactosamine	200
**3. *N*-acetylglucosamine group**			
Lycopersicon esculentum (tomato) lectin (LEL)	20	Chitin hydrosylate	200
Solanum tuberosum(potato) lectin (STL)	20	Chitin hydrosylate	200
Wheat germ agglutinin (WGA)	10	Chitin hydrosylate	200
Datura stramonium lectin (DSL)	20	Chitin hydrosylate	200
Griffornia simplicifolia lectin II (GSL-2)	20	No inhibitor used	
Succinylated WGA (SWGA)	20	Chitin hydrosylate	200
**4. Galactose group**			
Peanut agglutinin (PNA)	20	Galactose	200
Erythrina cristagalli lectin (ECL)	20	Lactose	200
Jacalin (JACALIN)	20	Galactose	400
**5. Fucose group**			
Ulex europaeus agglutinin I (UEA-1)	10	No inhibitor used	
**6. Oligosaccharide group**			
Phaseolus vulgaris erythroagglutinin (PHA-E)	20	No inhibitor used	
Phaseolus vulgaris Leucoagglutinin (PHA-L)	20	No inhibitor used	
**7. Sialic acid group**			
Sambucus nigra lectin (SNA)	10	No inhibitor used	
Maackia amurensis lectin II (MAL-II)	10	No inhibitor used	

**Table 2:** Primary and secondary antibodies used for the estimation of PRRs in MK1-OSU and SD-PJEC cells by flow cytometry.

PRR	Primary Antibody	Isotype Control	Secondary Antibody
TLR2	Mouse anti-human/mouse (ebiosciences)	Mouse IgG1 (ebiosciences)	Goat anti-mouse IgG Alexa Fluor 488 (Invitrogen)
TLR3	Mouse anti-human (ebiosciences)		
TLR4	Mouse anti-human (ebiosciences	Mouse IgG2a (ebiosciences)	
TLR5	Goat anti-human (Santa Cruz biotechnology)	Goat IgG (Santa Cruz biotechnology)	Chicken anti-goat IgG Alexa Fluor 488 (Invitrogen)
TLR6	Rat anti-human (ebiosciences)	Rat IgG2a, kappa (ebiosciences)	Mouse anti-rat IgG FITC (ebiosciences)
TLR7	Goat anti-human (Santa Cruz biotechnology)	Goat IgG (Santa Cruz biotechnology)	Chicken anti-goat IgG Alexa Fluor 488 (Invitrogen)
TLR8	Rabbit anti-human (Santa Cruz biotechnology)	Rabbit IgG (Santa Cruz biotechnology)	Goat anti-rabbit IgG Alexa Fluor 488 (Invitrogen)
TLR9	Rat anti-human (ebiosciences)	Rat IgG2a, kappa (ebiosciences)	Mouse anti-rat IgG FITC (ebiosciences)
MDA5, RIG-I	Goat anti-human (Santa Cruz biotechnology)	Goat IgG (Santa Cruz biotechnology)	Chicken anti-goat IgG Alexa Fluor 488 (Invitrogen)

**Table 3:** qPCR primers for the quantification of PRRs.

Gene	Primer Sequences	Annealing Temp (°C)	Accession Number
*TLR-7*	5′ACA ATG ATA TCG CCA CCT CCA CCA3′	55	NM_001097434
	3′TGG CCA AGG AGA GAG TCT TCA GAT5′		
*RIG-I*	5′TAT CCG AGC AGC AGG CTT TGA3′	58	NM_213804
	3′TGA AGT TTA GGG TTC TCG TTG CTG GGA5′		
*MDA5*	5′TGC CCT TTC CCA GTG GAT TAC TGA3′	58	NM_001100194
	3′TGT GTC CAG CTC CAA TCA GAT GGT5′		
*RPL4*	5′CAA GAG TAA CTA CAA CCT TC3′	57	XM_003121741
	3′GAA CTC TAC GAT GAA TCT TC5′		

**Table 4:** Percentage of cells expressing TLRs-2, -4, -5, -6, -8, and -9 in MK1-OSU and SD-PJEC cells 24 h after influenza virus infection as determined using a flow cytometer. Values represent the average for four to six experiments ± SE. There was no significant difference in the expression between the infected and uninfected cells.

TLRs	MK1-OSU			SD-PJEC		
	Uninfected	MN08	IA07	Uninfected	MN08	IA07
**TLR-2**	65.1 ± 3.5	64.5 ± 12.5	73.9 ± 13.7	56.5 ± 14.0	52.1 ± 11.1	57.2 ± 14.1
**TLR-4**	7.3 ± 1.5	6.6 ± 0.7	3.9 ± 3.4	17.2 ± 6.0	22.3 ± 8.7	21.5 ± 12.5
**TLR-5**	23.6 ± 10.2	28.7 ± 12.4	26.9 ± 12.5	25.8 ± 20.2	21.8 ± 11.3	27.5 ± 26.1
**TLR-6**	26.7 ± 21.3	22.3 ± 12.4	26.4 ± 19.0	66.8 ± 7.2	66.8 ± 10.8	67.4 ± 10.9
**TLR-8**	17.2 ± 2.8	10.1 ± 2.2	11.5 ± 4.9	4.9 ± 3.5	8.2 ± 2.3	16.5 ± 4.1
**TLR-9**	32.7 ± 13.3	46.1 ± 16.6	35.5 ± 21.1	33.8 ± 22.4	31.8 ± 22.9	37.4 ± 16.1
